# A phase 2, open-label, single-arm study evaluating the combination of pembrolizumab, lenvatinib, carboplatin and pemetrexed in patients with metastatic non-small cell lung cancer harbouring targetable genomic alterations who progressed on standard tyrosine kinase inhibitors

**DOI:** 10.1007/s10637-025-01589-6

**Published:** 2025-11-10

**Authors:** Hoi Wai Chan, Chris Chun Long Tse, Yu-Chung Li, Roland Leung, Gin Wai Kwok, Jeffrey Sum Lung Wong, Karen Hoi-Lam Li, Josephine Wing Yan Tsang, Cho Wing Li, Jenny Wai Yan Lo, Vikki Tang, Rina Hui, Thomas Yau, James Chung Man Ho, Joanne Wing Yan Chiu

**Affiliations:** 1 Department of Medicine, Queen Mary Hospital, University of Hong Kong, Pokfulam, Hong Kong; 2 Hong Kong United Oncology Centre, Kowloon, Hong Kong; 3 Centre of Cancer Medicine, School of Clinical Medicine, University of Hong Kong, Pokfulam, Hong Kong

**Keywords:** Lung cancer, Non-small-cell lung carcinoma (NSCLC), Epidermal growth factor receptor (EGFR), Tyrosine kinase inhibitor (TKI), Pembrolizumab, Lenvatinib

## Abstract

Patients with metastatic non-small cell lung cancer (NSCLC) harbouring targetable genomic alterations who progressed on standard tyrosine kinase inhibitors (TKIs) have limited treatment options, with platinum-based chemotherapy offering modest efficacy. We evaluated the efficacy and safety of a novel combination of pembrolizumab, lenvatinib, carboplatin and pemetrexed in this population. This phase 2, open-label, single-arm study enrolled patients with metastatic NSCLC with sensitizing epidermal growth factor receptor (EGFR), anaplastic lymphoma kinase (ALK) or c-ROS oncogene 1 (ROS1) alteration who progressed on standard TKIs. Patients received a combination of pembrolizumab, lenvatinib, carboplatin and pemetrexed. The primary endpoint was objective response rate (ORR) per RECIST 1.1. Secondary endpoints included progression free survival (PFS), overall survival (OS) and safety. Of the 24 patients screened, 19 were enrolled and included in the intention-to-treat population. Median follow-up time was 10.7 months (95% CI 4.4–11.9). ORR was 31.6% (6/19, 95% CI 12.6%–56.6%; all partial responses). Median PFS was 11.9 months (95% CI 4.3–not reached). Median OS was not reached. Most of the treatment-related adverse events (TRAEs) were grade 1–2, which occurred in 63% (12/19) of the patients. The most common TRAEs were hypothyroidism (31.6%), nausea (26.3%), neutropenia (26.3%), thrombocytopenia (26.3%) and anorexia (21.1%). The combination of pembrolizumab, lenvatinib, carboplatin and pemetrexed showed modest efficacy and manageable toxicity in metastatic NSCLC patients with targetable genomic alterations who progressed on standard TKIs. NCT04989322.

## Introduction

Lung cancer is the leading cause of cancer death in adults, with an estimated 1.8 million deaths worldwide in 2022 [1]. Non-small cell lung cancer (NSCLC) accounts for 85% of all lung cancers [2]. Patients with metastatic NSCLC harbouring genomic alteration of particular tyrosine kinases, notably epidermal growth factor receptor (EGFR), and rearrangements of the anaplastic lymphoma kinase (ALK) gene and c-ROS oncogene 1 (ROS1) gene, are treated with tyrosine kinase inhibitors (TKIs). As a first-line treatment, the response rates of TKIs are in the range of 60%–80%, and median progression-free survival (PFS) is 10–17 months [3–7]. However, acquired resistance inevitably develops, and platinum-based chemotherapy is a conventional next-line therapy, with a median PFS of only 4–5 months [8].

Immunotherapy alone is ineffective in NSCLC with mutation of EGFR or rearrangement of ALK and ROS1 [9, 10], but the IMpower150 trial demonstrated that combining the anti-PD-L1 atezolizumab with the anti-vascular endothelial growth factor (anti-VEGF) bevacizumab and chemotherapy improved PFS (hazard ratio [HR] 0.59) among patients with EGFR mutations [11].

Lenvatinib is a multi-target inhibitor of VEGF receptors and other oncogenic pathway-related receptor tyrosine kinases [12, 13]. In a randomized phase 2 study, single-agent lenvatinib compared with best supportive care demonstrated a superior improvement in PFS in heavily pre-treated patients with NSCLC (20.9 vs 7.9 weeks, *p* < 0.001) [14]. The majority of these patients (85%) had prior erlotinib or gefitinib.

At the time of design of the study, preliminary results from the LEAP trials evaluating the safety and efficacy of lenvatinib plus pembrolizumab demonstrated robust anti-tumour activity and durable responses across diverse tumour types with a manageable safety profile [15].

This phase 2 study was designed with the background of the above studies and the postulated synergistic effects from combining pembrolizumab, lenvatinib and chemotherapy. It is based on leveraging complementary mechanisms of action to enhance anti-tumour efficacy. Chemotherapy has direct cytotoxicity and anti-angiogenesis effect. It also induces immunogenic cell death, releasing tumour antigens to prime the immune system for pembrolizumab to activate T cells [16]. Meanwhile, lenvatinib acts on the VEGF and other pathways to reduce immunosuppression in the tumour microenvironment, enhancing immune response from the use of pembrolizumab [17].

In the study, the efficacy and safety of the combination of pembrolizumab, lenvatinib, carboplatin and pemetrexed were evaluated in patients with metastatic NSCLC post-TKI progression.

## Methods

### Study design

This was an investigator-initiated, single-centre, single-arm, phase 2 study in patients with metastatic NSCLC harbouring a targetable mutation who progressed on standard tyrosine TKIs. This study adopted a phase 2 Simon’s two-stage optimal design. Participants were recruited at Queen Mary Hospital, Hong Kong, from October 2021 to December 2023. The cutoff date for data analysis was 2 February 2024.

**Fig. 1:**
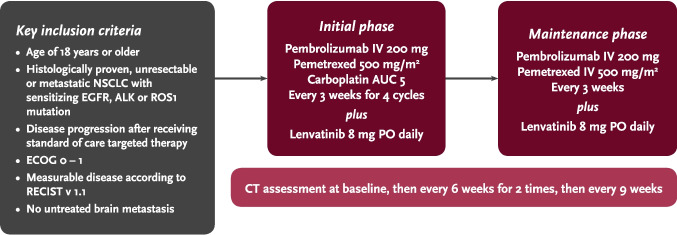
Key recruitment criteria and treatment regimen

During the initial phase, eligible patients were given intravenous (IV) infusion of pembrolizumab 200 mg every 3 weeks, pemetrexed 500 mg/m^2^, carboplatin at an area under the concentration–time curve of 5 mg/mL/min (AUC 5) every 3 weeks for up to 4 cycles and oral lenvatinib 8 mg daily. The treatment regimen is summarized in Fig. 1. After four cycles, patients with stable disease (SD) or responding disease were continued with maintenance pembrolizumab and pemetrexed every 3 weeks and daily dose of lenvatinib as tolerated until disease progression or up to 2 years (35 doses of pembrolizumab). Computerized tomography (CT) scans were conducted at baseline, every 6 weeks for two cycles, and then every 9 weeks. As per the investigator’s discretion, patients with unacceptable toxicity to pemetrexed at maintenance phase could go on with pembrolizumab and lenvatinib only. Treatment beyond progression was not allowed.

### Patients

The key inclusion criteria were an age of 18 years or older; histologically proven, unresectable or metastatic NSCLC with sensitizing EGFR, ALK or ROS1 mutation; disease progression after receiving standard TKI; Eastern Cooperative Oncology Group (ECOG) performance status score of 0–1; and measurable disease according to the Response Evaluation Criteria in Solid Tumors (RECIST), version 1.1. Patients with T790M mutation required prior osimertinib failure.

### Endpoints

The primary endpoint was ORR, defined as the proportion of patients who have the best response as a confirmed complete response (CR) or partial response (PR) per RECIST 1.1 at any time during the study. Secondary endpoints included PFS and overall survival (OS). PFS was defined as the time from initiation of treatment to the first documented disease progression per RECIST 1.1 or death due to any cause. OS was defined as the time from initiation of treatment to death from any cause or last follow-up date. Duration of response (DOR) was measured from the time a CR or PR was first documented to disease progression.

Patients were evaluated for adverse events (AEs) during study participation, and toxicity was assessed according to the Common Terminology Criteria for Adverse Events (CTCAE), version 5.0.

### Statistical analysis

Traditional chemotherapy yields a response rate of around 30% in patients who developed resistance to first line TKIs [4]. Response rate of 30% was therefore used as the benchmark for the study. The null hypothesis was that the combination would give a response rate of no more than 30%, and the one-sided alternative hypothesis was that the response rate would be no less than 50%.

Based on Simon’s two-stage design with 5% maximal tolerable false positive rate and maximum tolerable false negative rate of 20%, 15 assessable patients were enrolled in the first stage. If fewer than six patients responded, the study would be terminated, with the conclusion that the response rate was no more than 30%. Otherwise, up to 31 additional assessable patients would be enrolled.

The primary endpoint, ORR, was calculated as the proportion of patients achieving a response, with 95% confidence intervals (CIs) estimated using the exact binomial (Clopper-Pearson) method. A one-sided exact binomial test was performed to assess whether the ORR significantly exceeded 30%.

Time-to-event data, including DOR, PFS and OS, were estimated using the Kaplan–Meier method. The median DOR, PFS and OS were reported with 95% confidence intervals calculated using the Brookmeyer-Crowley method. The median follow-up time was estimated using the reverse Kaplan–Meier method.

Subgroup analyses, including ORR by PD-L1 expression and prior TKI lines, were conducted using the same statistical methods. They were exploratory due to small sample sizes.

All statistical analyses were performed using GraphPad Prism 10.

## Results

### Patient characteristics

**Fig. 2:**
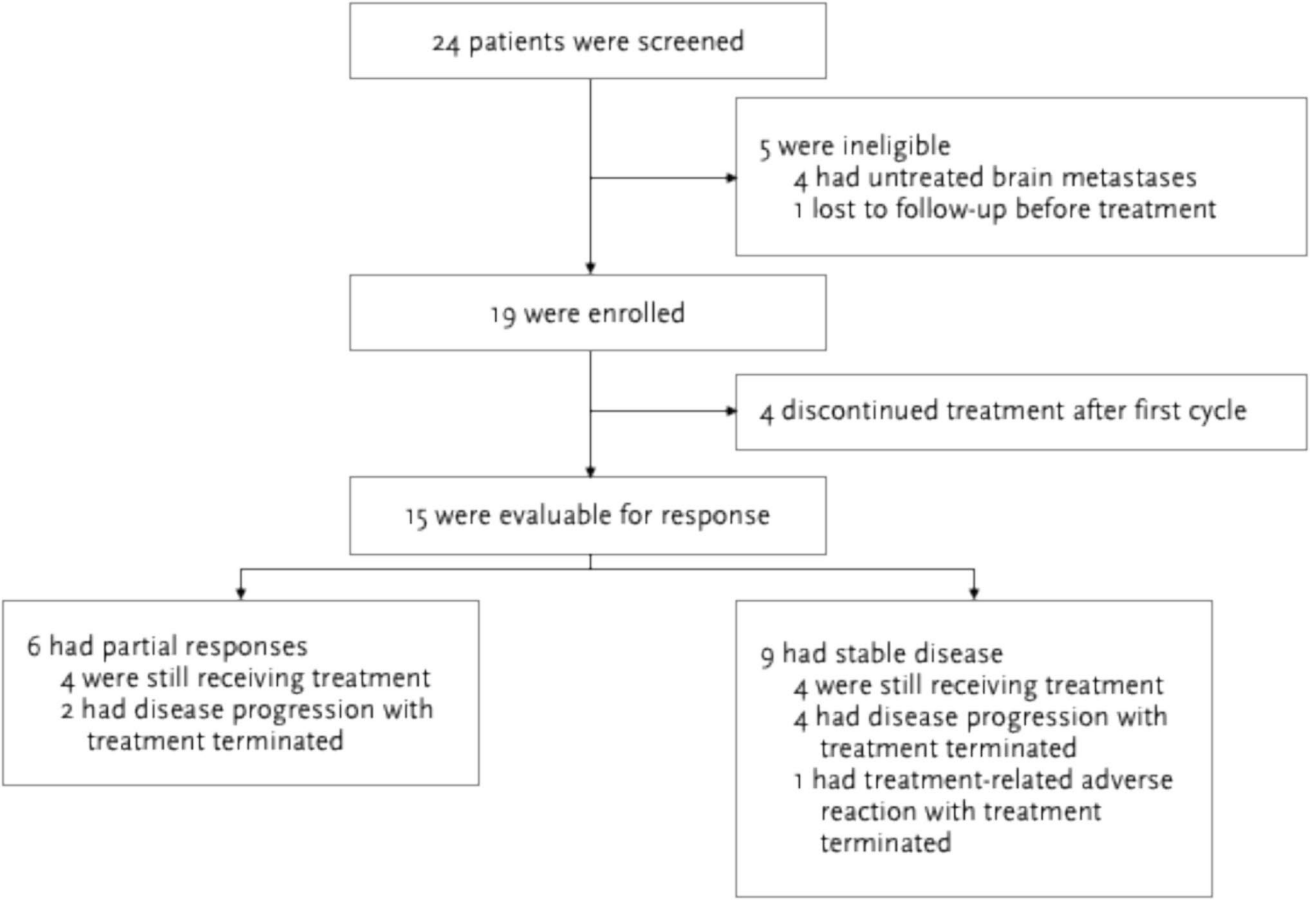
Study enrolment flowchart

Between October 2021 and December 2023, a total of 24 patients were assessed for eligibility, of whom 19 met the eligibility criteria and were enrolled (Fig. 2). All were ethnic Chinese from Hong Kong. Four patients were excluded due to untreated brain metastases, and one was lost to follow-up before study treatment was initiated. Four enrolled patients discontinued treatment after the first cycle due to admissions with acute medical problems unrelated to the treatment, including per rectal bleeding, pneumonia, pulmonary embolism and symptomatic brain metastasis with seizure. They were all included in the intention-to-treat (ITT) analysis but their responses were not evaluable due to lack of reassessment imaging. All enrolled patients were evaluated for safety.

Baseline demographics and disease characteristics of the enrolled patients are summarized in Table 1. Among the enrolled patients, the median age was 64 years (range 29–77 years). Ten (52.6%) were female. Six (31.6%) were current or former smoker. Five (26.3%) had treated brain metastases. All had biopsy-proven adenocarcinoma, 18 (94.6%) had tumours harbouring an EGFR mutation (exon 19 deletion, *n* = 6; exon 21 L858R, *n* = 10; exon 20 S768I, *n* = 1; exon 18 G719X, *n* = 1) and 1 (5.3%) harbouring an ELM4-ALK fusion. Fourteen (73.7%) received one previous TKI therapy, 4 (21.1%) received two previous TKI therapies and 1 (5.3%) received three previous TKI therapies.

**Table 1:** Demographic and disease characteristics of the enrolled patients at baseline

Characteristic	Enrolled patients (*n* = 19)
Median age (range)—yr	64.0 (29.0–77.0)
Male sex—no. (%)	9 (47.4)
Female sex—no. (%)	10 (52.6)
Race or ethnic group—no. (%)	
Chinese	19 (100)
ECOG performance-status score—no. (%)	
0	7 (36.8)
1	12 (63.2)
Smoking status—no. (%)	
Current or former	6 (31.6)
Never	13 (68.4)
Histologic features—no. (%)	
Adenocarcinoma	19 (100)
Known brain metastases (treated)—no. (%)	5 (26.3)
Sensitizing mutation—no. (%)	
EGFR mutation	18 (94.7)
Exon 19 deletion	6 (31.6)
Exon 21 L858R	10 (52.6)
Exon 20 S768I	1 (5.3)
Exon 18 G719X	1 (5.3)
ELM4-ALK fusion	1 (5.3)
ROS1	0 (0)
PD-L1 tumour proportion score (TPS)—no. (%)	
< 1%	4 (21.1)
≥ 1%	4 (21.1)
1–49%	2 (10.5)
≥ 50%	2 (10.5)
Could not be evaluated	11 (57.9)
No. of previous TKI therapy—no. (%)	
1	14 (73.7)
2	4 (21.1)
3	1 (5.3)
Previous TKI therapy—no. (%)	
Gefitinib	2 (10.5)
Erlotinib	6 (31.6)
Afatinib	2 (10.5)
Osimertinib	13 (68.4)
Brigatinib	1 (5.3)
Lorlatinib	1 (5.3)

### Efficacy

By the time of data cutoff on 2 February 2024, with a median follow-up of 10.7 months (95% CI 4.4–11.9) in the 19 patients in the ITT population, treatment was ongoing in 8 patients, 6 patients permanently discontinued treatment owing to disease progression, 1 patient permanently discontinued treatment owing to a treatment-related adverse event (TRAE) and 4 patients permanently discontinued treatment after the first cycle due to acute medical illnesses as described above.

The median duration of treatment was 4.8 months (range 0.3–20.4 months), 47.4% (*n* = 9) were treated for at least 6 months and 15.8% (*n* = 3) for at least 12 months.

**Fig. 3:**
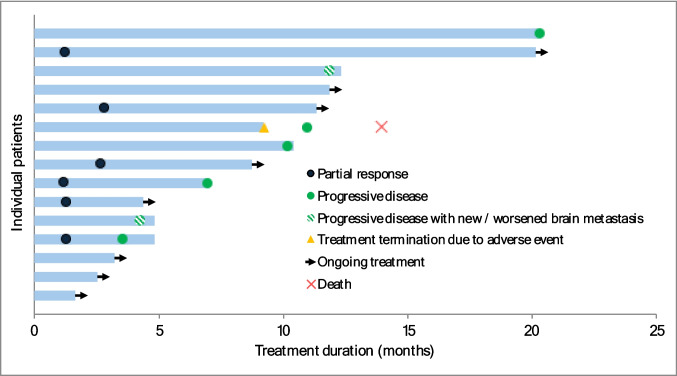
Clinical response during the study. Swimmer plot showing responses over time in 15 patients following the study treatment. Two patients had stable disease initially but developed progressive disease with new/worsened brain metastases. One patient discontinued from treatment due to an adverse event of immunotherapy-related nephritis

**Fig. 4:**
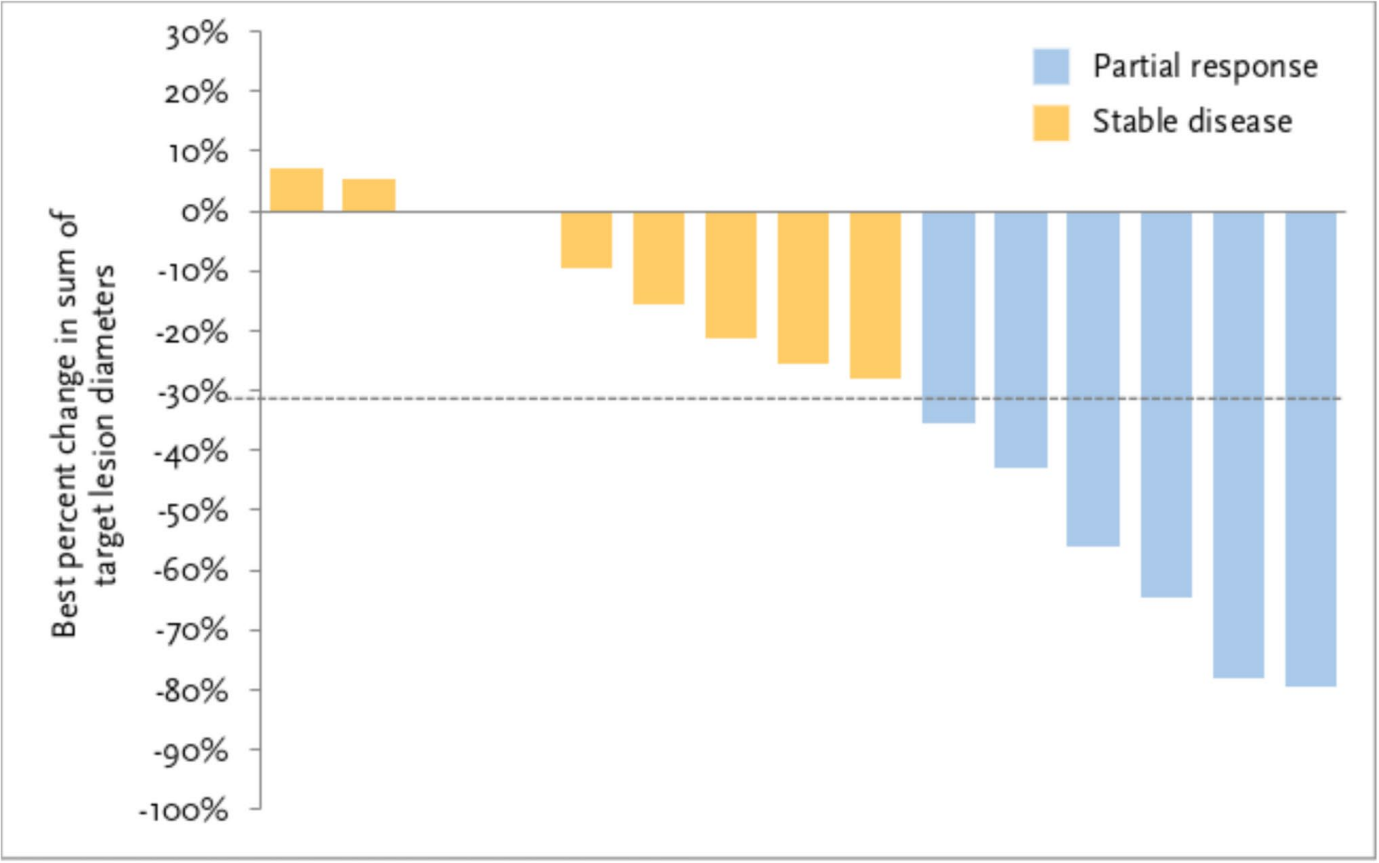
Tumour response during the study. Best overall response achieved by the 15 patients with evaluable responses. Change in tumour diameter between − 30% and 20% from baseline was considered stable disease, as per RECIST version 1.1. An increase of more than 20% in diameter from baseline was considered progressive disease, and a decrease of more than 30% in diameter from baseline was considered a partial response

**Fig. 5:**
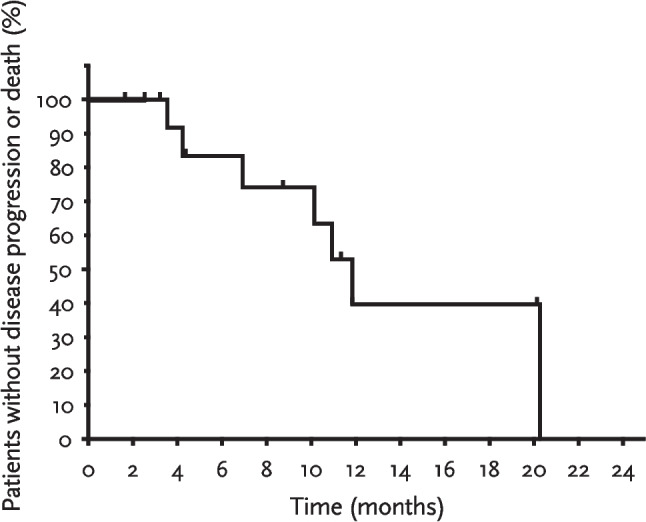
Progression-free survival. Kaplan–Meier estimates of progression-free survival in the 19 enrolled patients. Tick marks indicate censoring of data

ORR was 31.6% (6/19, 95% CI 12.6%–56.6%), with all six responses being partial responses (Fig. 3). The one-sided exact binomial test yielded a *p*-value of 0.55. Among the six responders, the median time to response was 1.3 months (range 1.2–2.8 months). Median DOR was not reached, with four patients censored at data cutoff. SD was observed in nine patients (Table 2). Two patients with SD eventually developed progressive disease with new or worsened brain metastases. Tumour response in target lesions assessed according to RECIST version 1.1 is shown in Fig. 4. The median PFS was 11.9 months (95% CI 4.2–not reached) (Fig. 5). Median OS was not reached with 14 patients censored at data cutoff.

**Table 2:** Summary of efficacy data

	Total population	PD-L1 expression			Previous lines of TKIs		
		TPS ≥ 50%	TPS 1–49%	TPS < 1%	3	2	1
Parameter	*n* = 19	*n* = 2	*n* = 2	*n* = 4	*n* = 1	*n* = 4	*n* = 14
Best overall response, *n* (%)							
CR	0 (0)	0 (0)	0 (0)	0 (0)	0 (0)	0 (0)	0 (0)
PR	6 (31.6)	2 (100)	1 (50)	3 (75)	0 (0)	1 (25)	5 (35.7)
SD	9 (47.4)	0 (0)	1 (50)	1 (25)	0 (0)	2 (50)	7 (50)
PD	0 (0)	0 (0)	0 (0)	0 (0)	0 (0)	0 (0)	0 (0)
Not evaluable^a^	4 (21.1)	0 (0)	0 (0)	0 (0)	1 (100)	1 (25)	2 (14.3)
ORR^b^, % (95% CI)	31.6 (12.6–56.6)	100 (15.8–100)	50 (1.3–98.7)	75 (19.4–99.4)	NE	25 (0.6–80.6)	35.7 (12.8–64.9)
Median DOR, months (95% CI)	NR	NR	NE	NR	NE	NE	NR
Median PFS, months (95% CI)	11.9 (4.2–NR)	NR	20.3 (NE)	5.6 (3.6–NR)	NE	NR	10.9 (4.2–NR)
Median OS, months (95% CI)	NR	NR	NR	NR	NE	12.8 (5.5–NR)	NR

*CR* complete response, *PR* partial response, *SD* stable disease, *PD* progressive disease, *DOR* duration of response, *CI* confidence interval, *NE* not estimable, *NR* not reached, *SD* stable disease, *PFS* progression-free survival.

^a^Including patients without post-baseline assessment on the data cutoff date.

^b^Objective response rate (ORR) was calculated based on the intention-to-treat (ITT) population and included PR and CR.

The efficacy data were further analysed according to the PD-L1 TPS and previous lines of TKIs received.

Two patients had PD-L1 TPS ≥ 50%. ORR was 100% (2 PR; 95% CI 15.8%–100%). Median DOR, PFS and OS were not reached. Two patients had PD-L1 TPS 1–49%. ORR was 50% (1 PR, 1 SD; 95% CI 1.3%–98.7%). For the patient with PR, DOR, PFS and OS were censored. In the patient with SD, PFS was 20.3 months and OS was censored. Median OS was not reached. Four patients had PD-L1 TPS < 1%. ORR was 75% (3 PR, 1 SD; 95% CI 19.4%–99.4%). Median DOR was not reached. Median PFS was 5.6 months (95% CI 3.6–not reached). Median OS was not reached. The other 11 patients had non-evaluable PD-L1 status.

One patient received 3 lines of TKIs. Response was non-evaluable. PFS was censored. OS was 1.2 months. Four patients received 2 lines of TKIs. ORR was 25% (PR 1, SD 2, NE 1; 95% CI: 0.6%–80.6%). For the patient with PR, DOR was censored. Median PFS was not reached. Median OS was 12.8 months (95% CI 5.5–not reached). Fourteen patients received 1 line of TKI. ORR was 35.7% (PR 5, SD 7, NE 2; 95% CI 12.8%–64.9%). Median DOR was not reached. Median PFS was 10.9 months (95% CI: 4.2–not reached). Median OS was not reached.

### Safety

Of the 19 patients in the ITT population, TRAEs of any grade occurred in 74% (*n* = 14) (Table 3). Most of the TRAEs were grade 1–2, which occurred in 63% (*n* = 12) of the patients. Grade 3 or 4 events were reported in 19% (*n* = 3) of the patients.

**Table 3:** Summary of treatment-related adverse events

Event, *n* (%)	Any grade	Grade 1	Grade 2	Grade 3	Grade 4
	**All enrolled patients (*n* = 19)**				
Any TRAE	14 (74)	10 (53)	12 (63)	2 (11)	1 (5)
Hypothyroidism	6 (32)	1 (5)	5 (26)	0 (0)	0 (0)
Nausea	5 (26)	2 (11)	3 (16)	0 (0)	0 (0)
Neutropenia	5 (26)	2 (11)	1 (5)	2 (11)	0 (0)
Thrombocytopenia	5 (26)	3 (16)	0 (0)	1 (5)	1 (5)
Anorexia	4 (21)	2 (11)	2 (11)	0 (0)	0 (0)
Hypertension	3 (16)	1 (5)	2 (11)	0 (0)	0 (0)
Anaemia	2 (11)	0 (0)	1 (5)	1 (5)	0 (0)
Pruritus	2 (11)	1 (5)	1 (5)	0 (0)	0 (0)
Rash	2 (11)	1 (5)	1 (5)	0 (0)	0 (0)
Abdominal pain	1 (5)	1 (5)	0 (0)	0 (0)	0 (0)
Adrenal insufficiency	1 (5)	0 (0)	1 (5)	0 (0)	0 (0)
ALT raised	1 (5)	1 (5)	0 (0)	0 (0)	0 (0)
AST raised	1 (5)	1 (5)	0 (0)	0 (0)	0 (0)
Diarrhoea	1 (5)	1 (5)	0 (0)	0 (0)	0 (0)
Malaise	1 (5)	1 (5)	0 (0)	0 (0)	0 (0)
Nephritis	1 (5)	0 (0)	1 (5)	0 (0)	0 (0)
Palmar-plantar erythrodysesthesia syndrome	1 (5)	0 (0)	1 (5)	0 (0)	0 (0)
Vomiting	1 (5)	0 (0)	1 (5)	0 (0)	0 (0)
TRAE leading to treatment discontinuation	1 (5)				

Treatment-related adverse events were considered adverse events classified as related, possibly related and probably related to treatment. Adverse events were reported with the use of the Medical Dictionary for Regulatory Activities, version 21.0, and graded according to the National Cancer Institute Common Terminology Criteria for Adverse Events (CTCAE), version 5.0

*ALT* alanine aminotransferase, *AST* aspartate aminotransferase.

The most common TRAEs were hypothyroidism (31.6%), nausea (26.3%), neutropenia (26.3%), thrombocytopenia (26.3%) and anorexia (21.1%). Grade 3 TRAEs were neutropenia in two patients, anaemia in one patient and thrombocytopenia in one patient.

The only grade 4 TRAE was thrombocytopenia, which was deemed related to chemotherapy and resolved after supportive transfusion and stopping chemotherapy. The same patient suffered from grade 2 nephritis related to pembrolizumab. She was discontinued from the trial because of this adverse event.

No treatment-related death was observed.

## Discussion

In this phase 2, open label, single-arm trial, we evaluated the efficacy of the combination of pembrolizumab, lenvatinib, carboplatin and pemetrexed in 19 patients with metastatic NSCLC harbouring a targetable mutation who progressed on standard TKIs. This regimen demonstrated modest activity, with an ORR of 31.6% (95% CI 12.6%–56.6%) in the ITT population. The median PFS was 11.9 months (95% CI 4.2–not reached), suggesting prolonged PFS benefit in some patients. The treatment was generally well tolerated, with most TRAEs being grade 1–2.

The ORR of 31.6% in the ITT population is close to the null hypothesis value of 30% and below the alternative hypothesis value of 50%. Given that the 95% CI includes 30% and the one-sided exact binomial test yielded a *p*-value of 0.55, the null hypothesis (ORR ≤ 30%) cannot be rejected. These findings suggest that the combination of pembrolizumab, lenvatinib, carboplatin and pemetrexed has modest activity in this patient population, comparable to traditional platinum-based chemotherapy. The prolonged PFS in some patients indicates potential benefit in selected individuals. Early termination of recruitment at 19 patients, rather than the planned 46, reduced statistical power, which in turn increased the chance of a false negative result and limited the ability to detect a clinically meaningful ORR improvement.

Exploratory subgroup analyses by PD-L1 TPS and prior lines of TKI showed variable ORRs (25%–100%). The small subgroup sizes (e.g. *n* = 2 for TPS ≥ 50%, *n* = 1 for 3 TKIs) resulted in wide CIs and unreliable estimates. These analyses, unadjusted for multiple comparisons, should be interpreted cautiously and considered hypothesis-generating only.

Brain metastasis posed a significant challenge in our group of patients. Among those with an EGFR mutation, 10 out of 14 of the patients were receiving osimertinib before enrolment into the study, while the one patient with ELM4-ALK fusion was receiving lorlatinib. Both osimertinib and lorlatinib are known to be highly penetrant into the central nervous system (CNS) with good CNS activity. In the six patients who eventually developed disease progression on the trial regimen, two had either new or worsened brain metastases, and they received osimertinib before the trial. Meanwhile, CNS progression was observed in only 6% of patients in the front-line osimertinib FLAURA trial [19]. Discontinuing TKIs may have reduced protection against CNS metastasis, accounting for the high proportion of patients developing new or worsened brain metastases in our study, suggesting inferior CNS activity of the study regimen comparing with osimertinib and lorlatinib. Some retrospective studies support the practice of continuing osimertinib post-progression with additional therapies [27, 28], but data from high quality prospective trials are needed to validate this approach.

This study had several limitations. There was a lack of a control group for comparison. The sample size was small (*n* = 19) and early termination limited statistical power. The small and homogeneous cohort of patients comprising all ethnic Chinese was not representative of the broader patient population and affected generalizability of the study results. In view of these limitations, our study findings require validation through larger, randomized trials.

The treatment of metastatic NSCLC after TKIs remains challenging. For EGFR-mutated NSCLC, despite a dramatic initial response to EGFR TKI, resistance generally develops after a median of 1 to 2 years [18]. With the use of a first- or second-generation TKI, half of the patients develop secondary EGFR exon 20 T790M mutation, for which a third-generation TKI such as osimertinib may be used [19]. Those who received a third-generation TKI as the first line treatment may develop mechanisms of resistance with MET amplification, HER2 amplification, ALK translocation, RET translocation and EGFR C797S mutation [20, 21]. Another subset of patients may develop histologic transformation to SCLC [22]. Platinum-based chemotherapy remains a standard option for patients who progressed after osimertinib or developed resistance to a first- or second-generation TKI not mediated by T790M, but the PFS is relatively short with a median of approximately 3 months only [23]. The combination of EGFR/MET bispecific antibody amivantamab with platinum-based chemotherapy has emerged as an additional therapy for this group of patients based on the MARIPOSA2 study [8]. However, the modest PFS gain from 4.2 to 6.3 months and the significant toxicities and high cost from amivantamab hinder the wide clinical application of this regimen. Another option is the combination of immune checkpoint inhibitors with chemotherapy, but there have been conflicting results in trials which included patients with an activating driver mutation [24, 25]. In the recently published HARMONi-A (AK112-301) study, ivonescimab, an anti-PD-1/VEGF bispecific antibody, plus chemotherapy significantly improved PFS (7.06 months vs 4.80 months with placebo) with a manageable safety profile in patients with NSCLC previously treated with EGFR TKI [26].

Our study was conceptualized after the results of the IMpower150 study were published in 2021. There was a significant delay in setting up the study and recruiting patients owing to the COVID-19 pandemic. After the initiation of this study, there were a number of new studies in the treatment of metastatic NSCLC using the same drug combination, in particular the LEAP-006 and LEAP-008 studies [15]. LEAP-006 was a randomized, placebo-controlled phase 3 trial evaluating pembrolizumab with pemetrexed and platinum-containing chemotherapy with or without lenvatinib as first-line treatment in metastatic NSCLC without existing EGFR-, ALK- or ROS1 genetic aberration. LEAP-008 was a randomized, open-label phase 3 trial evaluating pembrolizumab plus lenvatinib vs docetaxel as a second-line therapy in the same patient population who progressed on a PD-(L)1 inhibitor and platinum-based chemotherapy. Nevertheless, final analysis of both studies failed to demonstrate a significant improvement in the primary endpoints of OS and PFS and the secondary endpoint of ORR [29, 30]. These results are particularly noteworthy as both trials were part of a broader strategy to explore the potential of pembrolizumab and lenvatinib in treating various cancers, including NSCLC. The LEAP-006 study especially utilized a regimen that was similar to the one in our study. The lack of an overall benefit may possibly be due to the unique biology of NSCLC or insufficient tumour microenvironment modulation by lenvatinib, in addition to the increased toxicities with the combination, resulting in limited dose intensity and patient tolerability. Although there were meaningful clinical responses in some patients and our study met the threshold to proceed to the next stage, it was decided to prematurely close the trial in view of the negative results from the LEAP studies.

In conclusion, the combination of pembrolizumab, lenvatinib, carboplatin and pemetrexed showed modest efficacy in patients with metastatic NSCLC who had targetable mutations and progressed on TKIs. Further investigation with a larger sample size or alternative therapeutic strategies is warranted to enhance treatment outcomes in this challenging population.

## Data Availability

The datasets generated during and/or analysed during the current study are available from the corresponding author on reasonable request.
